# Principles of Early Mental Education

**Published:** 1859-07-01

**Authors:** 


### Art. III.—PRINCIPLES OF EARLY MENTAL EDUCATION.

In the last number of this journal, in an article on the artificial production of stupidity in schools, we sought to call the attention of our readers to the possibility that harm, rather than good, may frequently result from the powerful educational influences of the present day:—when those influences are either undesirable in their nature, or misdirected in their application.

Our argument was mainly intended to show, in the first place, that the variations witnessed among adults, in point of mental capacity, are far too considerable to be explained by differences in original conformation; and, secondly, that the tendency of the encephalon to automatic action may be regarded as the chief source of practical errors in the art of teaching.

We return to this subject, at the risk of being thought to expound a truism, because we are deeply impressed with its importance to the community. We believe that education can never be successfully and economically conducted, as regards the masses, until it is based upon sound physiology; and that empirical teaching, however, or from whatever cause, it may occasionally develope the powers of individuals, must always be wholly insufficient to afford secure foundation for a national system. The facts and arguments by which these propositions are supported, are of a kind to appeal with peculiar force to the members of the medical body; and hence our brethren, or at least those among them who have been led to reflect at all upon educational questions, are enabled to be in advance of, and are called upon to lead, the opinions of the general public. Outside the pale of the profession, it is still a matter of novelty, and even of wonder, that physiology should furnish rules for the preservation of bodily health; and that the wholesale violation of these rules should be followed by commensurate calamities. Official personages, from the departments of the War Office to a country board of guardians, from the country board of guardians to a village Dogberry, are almost unanimous in endeavouring to believe that the laws of vital action, as enunciated by medical authority, are only the crotchets of the individual doctor who may give them utterance; and that they may be disregarded without guilt, or forgotten without punishment. It is conceivable that the public mind may once have been in a similar position, relatively to the forces that govern inert matter; that architects may have striven to build in spite of gravitation; or shipwrights to defy the conditions of buoyancy. It is conceivable that, a century hence, a verdict of insanity would screen a martinet who might reproduce Crimean disasters, or await a constable who had connived at his neighbour's cesspool. But, in the present state of public enlightenment, it has yet to be realized that physical and vital forces are modes of the same agency, producing their effects, under known conditions, with equal and with unerring certainty. Still less is it generally known or admitted that the various actions which are called intellectual can be referred, each to its proper organ in the nervous centres, each to definite circumstances modifying the course of the impression in which it had its origin. On this, and on many other subjects, there is, we think, a tendency to credit intelligent persons with a larger amount of scientific information than they usually possess; and to expect from them more just opinions than they have ever been enabled to form. The editor of a provincial newspaper did us the honour to criticize in his columns our article on the production of stupidity; and to select our physiological heresies for special animadversion. We argued, he said, as if from a tripartite division of the central nervous system, and referred to a brain proper, a sensorium, and a spinal centre; whereas it was well-known that the central nervous system can be divided into *two* parts only,—the CORTICAL and the MEDULLARY! We transfer this exquisite *morceau* to our pages, not only because it is the most ludicrous blunder that ever was couched in language, but because it furnishes a clue to the value of popular notions upon all kindred subjects. If such be the ignorance of those who write our newspapers, how much knowledge may we expect from those who read them? The only possible answer must lead to the inference that the elements of physiology are not sufficiently comprehended by the public to be of general utility in guiding conduct or habits, even where bodily health alone is concerned; and, as our knowledge of the functions of the nervous system is not only somewhat intricate in itself, but is, moreover, among the latest triumphs of research, so, in this department, more conspicuously than in any other, it is the duty of the physician to diffuse abroad that light which, by reason of his elevated scientific position, falls first across his path:—when as yet its source has not risen high enough to dispel the clouds and mists which obscure the lower portions of the track, or to illuminate the gloomy caverns which are the lurking-places of prejudice and error.

There is, perhaps, no pursuit known among men that has occupied a larger share of time, employed a larger amount of skill, or been watched with a more absorbing interest, from the beginning of history to the present day, than the work of educating the young. Ambitious teachers, seeking their own fame through the reputation of their pupils; parents, noting the progress of their offspring with the most aspiring or the most affectionate solicitude; philosophers, bent upon discovering in what degree the intellectual development of the human race could be modified or controlled by cultivation; all alike have devoted their best talents, stimulated by their most ardent desires, to the solution of the great problem that surrounds the earlier years of life. It is not too much to say that all alike have failed. They have failed not only in obtaining any uniformity of result, not only in every attempt to define the limits of their own powers, but even in establishing upon satisfactory evidence that they possess, as regards the faculties of the mind, any powers whatever. The same household, the same school, the same college, may send forth the senior wrangler and the wooden spoon; leaving it an open question whether the difference depended on conformation or on training; and a still more open question whether the positions of the two men might not be reversed in after life. With regard to children, there is probably no professed teacher who would hesitate to promise that he would do what was best for each pupil entrusted to his charge; but there are very few who would attempt to predict what the results of that best would be. There is, apparently, no measure by which to ascertain the natural type upon which each brain is formed; and, consequently, no standard by which to estimate the effects of cultivation.

There is, it need hardly be said, an abundance of education, in the sense in which the word is used by Paley; namely, to express every preparation that is made in our youth for the sequel of our lives. The nature of this preparation has varied, and will vary, with the fluctuations of fashion and circumstance; but we may always learn from it that, several years being given to the task, and average capacity to the pupil, any desired attainments may be secured with tolerable certainty.

This much being granted, there remains the remarkable fact that the power to confer attainments is not the result of any direct control over the faculties of the mind. Of two boys at a public school, who had spent the whole period of youth in making verses, it might be found that the exercise, in one case, had served to call into activity the highest powers of the intellect; in the other, the exercise itself having still been tolerably well performed, these powers might be in comparative abeyance. The old explanation of this would be, that the brain of the latter pupil was sterile ground; but the case is too common for the explanation to be a sound one. In such instances, lamentably frequent even in the best schools, there is usually a certain relation between the length of time given to teaching, and the duration of the acquirements which result from it. Descending a step lower in the social scale, to classes who can neither command the best schools for their children, nor retain them for a very prolonged period under tuition, it may be observed that mental cultivation among the pupils becomes more exceptional; and also that their attainments are less permanent; while on reaching the level of elementary schools under inspection, or the lower depth of those, for pupils of the same rank, that are not under inspection, we find mental cultivation only in rare and solitary instances, and attainments so fleeting that they can scarcely be said to have any duration at all.

If this view be correct, and we entertain it as the result of careful and repeated inquiry, a doubt naturally occurs as to whether the schools devoted to the education of the lower middle, and labouring classes, serve, nationally speaking, any useful purpose. Persons now living have witnessed a great change in their character and management; but has this produced any concurrent change in their efficiency? The late Dr. Nott, tutor to the Princess Charlotte, used to relate that the "dame" of his native village taught her pupils to call every word of more than three syllables "*Nebuchadnezzar;*" while the certificated masters of the present day profess to pay especial attention to punctuation, meaning, and proper emphasis. But go into any elementary school, and subject the reading of the pupils to a real test, and there will not be found, we apprehend, one school in a hundred in which the advance on the Nebuchadnezzar system will be sufficiently important to deserve record, or to influence in the slightest degree the power of the pupils to read, or their pleasure in reading. Certain prepared passages may be pronounced aloud from an open book, in such a manner as to convey their sense; but unprepared passages, even of simple words, if they afford a single opportunity to blunder, will seldom indeed see the opportunity lost. As a rule, the eye and mind of the reader do not precede his voice; than which there can be no clearer proof that the meaning of the matter read is not taken into account. Ludicrous mistakes between words of somewhat similar sound but diverse sense, such as saying "mutton" for "motion," are of continual occurrence. Here the fault is in the eye, or rather in the optic ganglia of the sensorium, and the intelligence does not correct it, because not at all employed or concerned in the operation. We recently heard an error of an analogous kind, on the occasion of the admission of some youths into a benefit society. They were required to repeat after the secretary a prescribed formula, containing a promise, if they saw any other member committing a fault, "gently to apprize him of it, as becometh a brother of this order." They all said, "gently to praise him for it." They had all recently been pupils at what is called a "good" elementary school, and they were all alike incapable of ascending to the height of an intentional paraphrase. It is difficult, however, or hardly possible, to ascertain the presence or the lack of general improvement in the art of reading, because the materials for comparison do not exist. Of course, more children are taught to read now than formerly; many more relatively to the population as well as absolutely; but the question is, whether they are so taught as to derive any advantage, any really useful and available knowledge, from the teaching? "It must be strange," writes Mr. Dickens, "to shuffle through the streets, unfamiliar with the shapes, and in utter darkness as to the meaning, of those mysterious symbols, so abundant over the shops, and at the corners of the streets, and on the doors, and in the windows! To see people read, and to see people write, and to see the postmen deliver letters, and not to have the least idea of all that language, to be, to every scrap of it, stone blind and dumb!" This is a striking picture; but is the condition of the man to whom every long word is Nebuchadnezzar, and in whose mouth the sound Nebuchadnezzar represents no idea, really much in advance of what the novelist describes? Is the sensational knowledge of an artificial connexion between certain symbols and certain sounds,—a knowledge not only unconnected with the intelligence, but almost antagonistic to it, a sufficient end to be gained by the annual expenditure of nearly a million sterling, by the constant labour of Privy Councillors, inspectors, teachers, children? The so-called "reading" is often the only attainment afforded by the elementary school that is discoverable a few months after the removal of the pupils; and any one who will be at the trouble of inquiring, may soon be satisfied as to its precise value.

*Litera scripta manet.* In the art of writing, the works of the past and of the present are alike open to our observation; and it is not difficult to ascertain how much improvement (as regards the results of teaching, not the number of people taught) has been produced by educational grants and Minutes in Council. We have before us two letters, written, with an interval of nearly a century between them, by persons in humble life, whose families were tenants upon the same estate, in an agricultural county. There is a great difference in the subjects of which they treat; the first having reference to a dispute about possession of a garden, and the second being from a young soldier, describing his experiences of recent Indian warfare. But the extracts we give will suffice to show that the teaching of former times is not greatly surpassed by that of the present day, in so far at least as the power of the pupils to write intelligibly is concerned.

The correspondent in the eighteenth century writes:—

"I shoud be a blicht to your Onear wud writ to the Weddear —— Con sorning the Gardon for she ont Let me Gardeney. * * * * * I Shoud Be Glad if I Ded now wen that man oud be to —— a bout the Repears most Be Don soun for Spreng oul Draw a Long * * * * Ples to Let me now wot Day Hegh oul be thear Be Caus I Lev at a destenc I mit not be thear els with out I now the de of his coming * * * * Ples to zend me ancear Dey Reckley and if you zend a leter to the Wedear I can com to hor."

The Indian soldier writes:—

"—— wee march fus marches til wee joind head Quarters of our Armey on the 18 small body of the Enemey advanced on our head Quarters the com in con tack with each uther the fout about 3 hours wee was not soon anaf for the fit wee was on day to Leat. We coud hear the begg Guns the fout verre hard for the tim it Leas * * * * wee was forcs to fit her an thear all nit we was faust to keep sheften our pesshen for our Enemey keep letten oup blu Lits all the night that the coud see as clar as Day for 3 mils rown soon as the see us the fir of all their beeg Guns at us than wee was fus to shef our pesshen agen our Deer Commerats was Lien ded all rown us * * * * our Enemy mead thar retreat wen the seed the British infintary and cavalry commin ful charg fess to fess the mead thar retreat the did not lik to see the Bennett glezsen in their fess * * * * our tilleary wos fus to fier blaink their ball Aminshen was Don for to mak the Enemey think the plenty * * * * I ward my sheart 2 months with thout washin when i took him of for to wash him her falld in pesses than i went with thout shert," &c., &c., &c.

The young soldier's letter is of considerable length; and, in many passages, displays considerable descriptive power. Our extracts from it show errors of precisely the same character as those in the letter written nearly a hundred years before. In both cases they are the errors of men not much accustomed to write, and not in the habit of reading; but who endeavoured to find means of expressing their ideas upon paper. We may safely conclude that writing was a laborious task to them both, only undertaken under the pressure of a strong motive; and also that reading was certainly not among their pleasures. Had it been so, they would have been as familiar with the sight as they were with the sound of the words they wished to use; and could not have fallen into ludicrous mistakes through trusting to their ears for guidance. The effect of such guidance is conspicuously manifest, in the first letter, in "Dey Reckley;" and, in the second, in "Commerats" and "Bennet." In a descriptive passage, not cited above, the writer states that his regiment did great "exqushn:" an attempt to set down what was colloquially familiar to him. In both letters, dim memories of words once seen in print, appear to obscure the simple phoneticism that is the manifest ruling principle; and of this the correct spelling of "head Quarters" (words often before the eyes of the soldier) furnishes a good example. On the whole we think it is fair to conclude that the comparatively modern school, equally with the comparatively ancient one, had failed to confer the power of reading,—of reading unconsciously, that is, the mind being occupied about the ideas or information conveyed by the composition, and not upon the mere deciphering of the words themselves. If children are taught to *read,* in the proper acceptation of the term, they will inevitably like reading, will become engrossed by it, and will seize eagerly upon all books within their reach. If they are only taught to *decipher,* they will find the labour irksome; and, when freed from restraint, will seldom or never practise it. In the former case they become so well acquainted with the appearance of words that it would not be possible for them to commit very grave errors in spelling; while, in the latter, the unaided ear may lead them astray through all possible permutations of the alphabet.

We have selected these two acquirements, reading and writing, as the subjects of the present article, not only because they are common to all schemes of education, nor entirely because the plan upon which they are taught will often, we believe, determine the character of mind of the learner, but in some measure because the actions performed are sufficiently simple to be easily analysed and tested, and referred to the organs concerned in producing them. Reading and writing *may be* so taught as to stimulate the intellectual faculties, and to keep the sensorium in its duly subordinate position. They often *are* taught (not only in the humble dame school, where writing is the Ultima Thule of the educational chart, but) even by those whose province it is to instruct the budding minds of hereditary legislators, in a manner that stimulates, or even morbidly excites, the sensorium alone, while it leaves the intellect torpid and unexercised. *Ce n'est que le premier pas qui coûte;* and a habit of sensational learning, acquired in the nursery, may be strong enough to baffle the best efforts of a teacher: the apparent progress of the pupil being, in reality, only a constantly increasing divergence from the path along which he ought to travel.

The specimens of correspondence that we have already laid before our readers are taken from the letters of persons in the rank of peasants. To show that the errors they committed are not peculiar to any class, either of schools or learners, we have yet another example of the epistolary art. The original was written by a young gentleman in the seventeenth year of his age, one nearly related to the possessor of an earldom, not labouring under any discoverable natural deficiency, and who had enjoyed all the advantages commonly attendant upon his social station. The occasion was the arrival of the writer at a fresh school, the master of which requires, from each pupil, upon joining, a letter that may serve as a test of his capacity, and as a starting point from which to estimate his progress. We need only observe further that the explanatory foot notes are not conjectural; but that they rest, in every instance, upon the authority of the author.

——— Schol,
Januaryn 24th, 185–.

Dear Sire,—The last week I spent the hollidays starern Eukerler[a] and history the first to or three reans[b], And the other part of the hollidays I spent in shouting[c] and driving.

At ——[d] I youst too learn history gorifea[e] a leate of latan[f] and dronge[g] and comperchin[h] and *earifme* reafmetic[i] somtines tables in the morning before becface in Sunday we use to go to church ones in the day then we yuesd to have diner at harepast one then from 3 to 4 we used to easemen[j] and read ore seat dawn till[k] oding[l] nothin at ole if we liked then we used to have teed at five then after we used to take wake[m] a noure and harf then when we came we used to have svav[n] in one of the roomes.

I reamin dear Siere
Your abeadint puple

---

The last few weeks, it may be observed, have placed before the public evidence from which we may infer that bad spelling is the rule, rather than the exception, among the rising generation in the upper section of the middle classes. We have ourselves met with many flagrant examples of it, but tried to encourage the hope that such cases were in reality unusual, and that their coincidence under our observation was accidental. The Civil Service Commissioners, however, have been told that a fault so trivial must interpose no obstacle to success at the pass examination for certain much-coveted appointments in the Government service; and my Lord Malmesbury is reported to have affirmed that bad spelling, although ungraceful and unbecoming, is not a proof of ignorance. It is, therefore, possible that the pigeon-holes of the Foreign Office, if they could be ransacked by profane inquirers, might furnish curiosities before which our humble illustrations of the hetero-graphic art would lose their piquancy and interest—specimens of hardy and exuberant cacography, now, alas! for ever hidden from the light of day—

> "Full many a gem of purest ray serene
> The dark unfathomed caves of ocean bear.
> Full many a flower is born to blush unseen,
> And waste its sweetness on the desert air."

In spite, however, of the high authority of my Lord Malmesbury, we see no reason to doubt that incorrect spelling is clearly a proof

[a] Studying Euclid. [b] Reigns. [c] Shooting.

[d] This blank was filled, in the original, by the name of a distinguished educational establishment: one that we should fear to mention, save with bated breath.

[e] Geography. [f] A little Latin. [g] Drawing. [h] Composition.

[i] Arithmetic. The letters in Italics show the primary conception, and were erased by the author.

[j] Assemble. [k] 7, understood. [l] Or doing.

[m] A walk. [n] Service—*i. e.* Divine Service.

Lessen of ignorance—ignorance of books, that is, in the manner we have already pointed out. There may possibly be exceptions. There may possibly—we will not say certainly or probably—be persons so organized that the forms of familiar words make no impression upon their consciousness; but all ordinary individuals, who are accustomed to read, learn to know words by sight, and would see an error in spelling, just as they would see errors in a picture inaccurately copied from an original that was well known to them. If boys or men are accustomed to read, they will, we repeat, be able to spell; and, *vice versâ*, if they cannot spell, it will be a fair inference that they are not accustomed to read. Moreover, there is a love of knowledge, inherent in all minds, that will induce the vast majority of people to read, to the very extent of their opportunities, upon subjects congenial to their respective tastes—unless the task of mere deciphering be so irksome and laborious as to outweigh the pleasure obtainable from the passages deciphered, or unless the inherent love of knowledge has been quenched at its source. We apprehend that both these contingencies are constantly occurring results of existing methods of teaching—methods opposed to the physiological laws which govern the action of the mind and nervous centres, and not less absurd, in relation to the ends proposed for attainment, than the attempts that have been made, from time to time, to act in defiance of the physical laws that control the universe. Even these physical laws, long as they have formed part of the stock of human knowledge, are often forgotten, or disregarded, until they vindicate their supremacy; and speculators will doubtless again be found, as they have been found aforetime, ready to expend their thoughts and capital upon cumbrous machines for aërial navigation, or to support impostors who profess to walk upon water by the aid of buoyant shoes. It cannot, therefore, be made matter of wonder, either that would-be educators are frequently ignorant of the principles upon which repose their chances of success, or that they often fail ignominiously in the task that with so much presumption they attempt. We think, however, that for the future such failures should be exhibited in a constantly decreasing ratio. We think it is the bounden duty of the medical profession to bring the principles which should regulate mental training prominently before the notice of the public, and to strive for their embodiment in precepts that may be rendered familiar to the humblest instructor. As a single effort in this direction, we purpose to inquire what physiological functions are involved in the reception of elementary teaching; how far the methods commonly prevalent are in harmony with those functions; and, where the harmony is wanting, by what means it can be ensured.

It may be stated, *in limine*, that the act of reading is of a somewhat complex character. It is performed by means of visual sensations, excited by certain symbols or words, which sensations may be the subjects either of volitional or of automatic attention, and may be associated either sensationally with the sounds, or intellectually with the ideas, that commonly belong to them. The first impression, however, is not necessarily visual, but may, as in the blind, be communicated through the organ of touch; and the association of the symbols with perceived or remembered sound, although general, is not essential, being absent in cases of deaf mutism, and in the many instances of persons who have acquired by the eye a book knowledge of some foreign language. It is commonly supposed that the visual organ must analyze each word into its component parts or letters, and that adult readers are constantly deciphering the page in this way, although habit may render them unconscious of the operation, and able to perform it with great rapidity. With this view we can by no means coincide, believing that all words, not of extraordinary length, are perceived as distinct objects, without any necessity for analysis, just as a friend is recognised at a distance, not by any observed combination of several peculiarities, but by the *tout ensemble* with which we are acquainted. Indeed, to those who read daily, common words are more familiar objects than detached letters; and it is a well-known fact that persons, who are doubtful about a question of spelling, will often write down both the alternatives that suggest themselves, so as to see, by comparison, what arrangement will produce the accustomed appearance of the desiderated word. Here, instead of the letters leading to the word, the word leads to the letters; and it would not be difficult to adduce many other illustrations of the same general principle.

We may regard every word, then, as being an object of visual sensation, associated either with a sound or with an idea, or with both. There is, probably, nothing to prevent the action of the cerebrum from taking place simultaneously with that of the sensorium, in immediate response to the impression; but it is impossible not to perceive that, in many kinds of reading, the cerebrum remains entirely quiescent, and the visual sensation is associated with a sound only. Physiologically speaking, the impression does not *pass on* to the cerebrum, but is reflected through the sensorium, and is exhausted in the production of articulate language. Such was the case in the school described by Dr. Nott, where, when the children *saw* a long word, they *said* Nebuchadnezzar; and such is the case, probably, in many much more pretentious establishments. We were once shown a copy of a sermon, printed, towards the end of the last century, in a country town in the west of England, and containing a very curious error. The frequently-recurring word *Almighty* had been abbreviated in the MS. thus—$A^{lty}$; the abbreviation was mistaken for $A^{tty}$, and the word was set up and printed, page after page, *Attorney*. It is clear that the compositor had not employed his mind about his work, and that his actions were consensual only.

The physiological causes which determine the reflection of the visual impression through the sensorium may be sought either in that organ itself, or in the cerebrum, and may be stated somewhat as follows :—

The sensorium may be unusually active, from:

- α. Natural vigour and acuteness.

- β. Artificial excitation.

The cerebrum may be torpid, from:

- α. Natural hebetude.

- β. Neglect, or want of stimulation.

- γ. Temporary exhaustion, by exercise, of the power of volitional attention.

- δ. Preoccupation by a train of thought.

With reference to these several conditions it may be observed that, while all impressions received by any of the senses appear to act as natural stimulants to the sensorium, and to call it into spontaneous or instinctive activity, they have not all the same tendency to pass on to the cerebrum. The proper excitants of the latter are probably the perceptions which, although of course communicated through the senses, have their origin in the distinctly intellectual operations of another individual; so that the mere visible symbols on a page, or the mechanical teaching of a master whose mind is not in his work, are sights or sounds addressed to the sensational instrument, and hardly at all to the intelligence. The frequent repetition of such sights and sounds, in obedience to the ordinary law of nutrition, must increase the energy of the organ they excite; and must depress, or relatively diminish, that of the organ they leave quiescent: from whence it follows that the brain of a child may soon grow into a settled habit of sensational action. The cerebrum, too, like every other apparatus that is subservient to the will, becomes fatigued, especially in its immature condition, much more speedily than the centres of automatic action; and hence it may frequently happen that the first portion of a lesson is understood; while the rest, failing to excite the wearied brain, is remembered through the sensorium as a matter of sights and sounds :—that is to say, of mere symbols, as distinguished from the ideas that the symbols were intended to convey. Pre-occupation by a train of thought, or what is called "absence of mind," is a condition not at all uncommon in connexion with the emotions of childhood; and it may exist, in a degree sufficient to impair the receptivity of the intellect, without at all diminishing the keenness of the senses. Such abstraction, as a result of volitional mental effort, is only witnessed in adult age; but a child may be intently thinking about the expected pleasure, or the dreaded discovery, of the morrow, without in any way losing his power to "learn" a lesson, and to repeat its sounds correctly.

From such considerations it would appear not difficult to frame the principles that should regulate all endeavours to instruct the young. The use of the sensorium is indispensable; but its abuse has to be guarded against: while the cerebrum requires to be trained to the gradual exercise of its powers. In order to fulfil these indications it is manifest that mere symbols should not be multiplied unnecessarily; and that time and attention should not be given to the attainment of proficiency in repeating sounds, or copying signs, to which no meaning can be attached. As soon as a written symbol is pointed out, and associated with a sound, the greatest care should be taken to connect the combined sensational impression with an idea, and to make the idea the leading feature in the combination: so that the sensorium may be used throughout as a feeder of the intelligence, but never encouraged to act as an independent organ. Lastly, if only one word had been learned, the first sign of weariness should conclude the lesson. The only healthful stimulus to application, in the case of a young child, is the pleasure that attends on the exercise of a new power; and this pleasure cannot strive against weariness, by whatever cause it may be induced.

Let us now take a common primer, and see how these principles are reduced to practice. A child is first taught to know, by sight and sound, the twenty-six letters of the alphabet, in their forms as capitals; these forms being generally obscured and complicated by pictorial illustrations. A highly coloured ox occupies the middle of O; and a flock of zebras form a pleasing background to Z. The next step is to connect the same sounds with the visible forms of small letters; and, when this task is accomplished to the satisfaction of the teacher, the pupil may expect to be advanced to such combinations as the following:

| | | | |
|---|---|---|---|
| ab. | ba. | ad. | da. |
| eb. | be. | ed. | de. |
| ib. | bi. | id. | di. |
| ob. | bo. | od. | do. |
| ub. | bu. | ud. | du. |

Before reaching za, ze, zi, zo, zu, it is obvious that many pages have to be wearily conned, that much time and patience must be expended, that frequent rewards or punishments will be required as antidotes to lethargy. In process of time, the learner is considered to be perfect in this portion of his training, is allowed to enter another circle, and finds there,—let us say:

| | |
|---|---|
| ban. | nab. |
| ben. | neb. |
| bin. | nib. |
| bon. | nob. |
| bun. | nub. |

And so on, through many similar arrangements, before he is brought face to face with real words of one syllable.

The whole course of the proceedings here faintly shadowed forth is, it need hardly be said, in direct opposition to the physiological principles we have endeavoured to lay down. From great A to zuz (if that combination be found in primers), the performance must be purely sensational (there being no ideas in any way related to the sights and sounds concerned); and a source of utter weariness to the pupil, as it is, unquestionably, to the teacher. In the first place the names of the letters have no relation to the sounds which these letters represent when joined; and, therefore, the power to repeat the alphabet, and to recognise the symbols composing it, are not in any degree steps towards the acquirement of the power of reading. The names of A and D being given, it would be impossible to excogitate from them the pronunciation of "*ad*;" so that the great and small alphabets consist simply of twenty-six aural and fifty-two visual sensations, with which no intellectual ideas can, during childhood, be associated, and in the "learning" of which no intellectual activity can ever be displayed. Pretty pictures make the matter ten times worse; first, by complicating the visible symbol; secondly, by giving rise to ideas that have no proper connexion with the matter in hand. In the mind of a child who is still learning the alphabet, there can be no right conception of the value of the initial letter of a word; and, consequently, no conception of the relation which the sign A bears, either to the sound of "archer," or to the picture of a gentleman in green, having a bow in his hand. Either the child accepts the connexion as a matter of fact, and remembers, without reflection, the three sensations presented to him; or else he forms some erroneous idea of the reason why they should be associated together. Furthermore, it is unquestionably true, as a general principle, that a butcher will often be the possessor of a "great dog;" but there is no sufficient reason why this fact in social science should be impressed upon the mind of young England in immediate sequence to the name and outline of the letter B. The reason of the case, indeed, is entirely opposed to such a procedure; because the proper aim of the teacher would be to avoid placing before the child any sight or sound which cannot be immediately connected with an intelligible idea: that is to say, any which is devoid of meaning, or any of which the meaning is above the grasp of infantile comprehension. The primer, so far as we have followed it, departs from this aim systematically; and presents to the learner page after page of vocal and visual combinations which exercise the sensorium alone, and exercise it the more actively, the more they are coloured, illustrated, and multiplied. During the whole process of hearing, seeing, imitating, and recognising them, the cerebrum cannot, in the nature of things, ever be brought into play; and hence, so far as we have gone, we find provision made to produce sensational activity, and to insure intellectual torpor.

When the reading lesson, properly so called, does at last begin, and the pupil is allowed to attempt real words of one syllable instead of sham ones, there are two points especially worthy of remark. In the first place much of what has been already learned must be forgotten or neglected; and, in the second, the methods in common use yield a prodigious number of merely aural impressions, without any corresponding ideas to be associated with them. For example, when the young reader meets with the verb *do,* he must blot out of his recollection that he has been taught, on a former page, to pronounce *do* as it is pronounced in the gamut; and a similar difficulty will meet him at almost every turn. In the ba, be, bi, bo, bu, combinations it is usual to give each vowel its natural or nominal sound; while, in actual words, the five vowels have, between them, as many as twenty-nine sounds, all of frequent occurrence. The supplementary or mute letters, too, of many words, will be sources of confusion to the learner; the y, for instance, in bay, or day, not changing the sound already given to *ba* and *da:* while an early lesson will be sure to contain something about a sheep, and to spell its bleat by the letters Ba. From all this it follows that a knowledge of the names of the letters, and a knowledge of ba, be, &c., are not only useless, but pos[illegible]ively hurtful and misleading, and continual sources of errors tha[illegible] require to be rectified by precept.

The multiplication of sensational impressions, without ideas to counterbalance them, depends chiefly upon the custom of making children spell words before they utter them. In saying *d, o, g, dog,* the pupil has to reproduce four sensations for one intellectual idea; while the sounds *d, o, g,* do not in any way lead to, or produce, the sound *dog;* so that the right order of succession among the four has to be remembered by the aid of the sensorium.

We have now, we think, succeeded in showing that the method of teaching a child to read, as usually practised, might not unfairly be described as a series of contrivances for promoting sensational, at the expense of intellectual, activity; or, in other words, for leading the pupil to neglect everything that he ought to do, and to do everything that he ought to avoid. As far as the art itself is concerned, there can be little question that the results obtained are as bad as bad can be. Most persons could count upon their fingers all the good readers among their acquaintance; and the performance of children is seldom other than a torture, to any one capable of understanding the meaning of written composition. It is trite to remark that ministers of religion fail, not unfrequently, in giving expression to the dignity and pathos of Holy Scripture; and it may be feared that they fail occasionally, even in giving expression to the evident meaning. It has been justly observed that an explanation of these familiar facts must be to be found, either in the surpassing difficulty of the art of reading, or in the exceeding faultiness of the principles on which it is taught; and it is hardly necessary for us to point out that there is a vast preponderance of evidence in favour of the latter solution of the difficulty. Our purpose is rather to maintain that the mischief done by bad methods of teaching is not limited to the production of bad readers; but extends, more or less, to every mental operation of the persons influenced. We think that such methods give a distinct impulse to sensational action at a period of life when the encephalon is being moulded to the manner of activity which will become habitual to it; and that the powers of the cerebrum will be passively diminished, in proportion as those of the sensorium are actively increased. In the present state of knowledge, we have no measure of the extent to which the natural balance between the various functions of the encephalon may be disturbed by educational influences; but we have reason to suppose that this extent is very considerable. Such influences are, in fact, or at least soon become, habits of mind in the children subjected to them; and it is impossible to doubt that a habit of attending to mere sights and sounds, and a habit of neglecting meaning, would have the strongest tendency to weaken the volition and the judgment, and to place them under the control of the centres of automatic action.

Our readers are entitled to ask, in what way these evils can be obviated; and, as far as method is concerned, the reply is easy. Mr. Baker, of Doncaster, has written, and Mr. Herbert, of Nottingham, has lectured, on the facility with which children learn to read, when the alphabet, and the unmeaning combinations of two letters, are altogether omitted from the scheme of instruction. The last named gentleman advises that the pupil should be shown printed words, in ordinary type, should be made to look attentively at each word as a whole, and to repeat it correctly after the teacher. He advises, also, that the daily lesson should not exceed ten minutes in duration; and he has found that, in this way, a child may be taught to read well, that is to say, with comprehension, good pronunciation, and proper emphasis, in a period varying from five to sixty hours; and, therefore, at the rate of ten minutes *per diem*, spread over the same number of weeks. The time most commonly required is from eight to ten weeks; and the sixty were occupied in a solitary instance. Mr. Herbert speaks of some of his pupils who, at the age of ten or twelve years, reading and writing well, and having acquired considerable proficiency in various branches of knowledge, had never learned the alphabet, and were unable to repeat it; and, as a practical question, he adduces evidence that is quite conclusive with regard to the excellence of the method that he recommends. The point of view, however, from which we are desirous to regard all methods of elementary teaching, has reference to their probable effect upon the mind and the nervous centres. It is evident, we apprehend, that the plan described by Mr. Herbert—the plan of making a word, instead of a letter, the unit of the system of reading, may be employed in such a manner as to remove all tendency to undue sensorial exaltation. In the first place, the words selected for the lesson should be those which represent ideas to the mind of the individual pupil, and should therefore be varied, in order to suit local or personal circumstances. There is, probably, no child, young enough to be learning to read, who attaches any distinct idea to "archer" or "zebra;" but any child, from five years old and upwards, will have distinct ideas of the meaning of the names of many domestic animals, many articles of dress, furniture, and household use, many trees, plants, and flowers, and also of the meaning of many verbs, adjectives, pronouns, and conjunctions. This knowledge, we conceive, should be utilized in the early reading lessons; and every word pointed out as an object of visual attention should already, by virtue of its sound, be an exponent of positive knowledge. When this is the case, the sensorial impression will pass on, and excite cerebral activity; or, if it should fail to do so spontaneously, the teacher may produce the desired effect by the aid of suggestion. In a farm-house, for instance, if the sentence "I see a cow," were taken for the first lesson, each word would strike home to the mind, would call up familiar ideas, and would initiate a source of pleasure in the power to recognise the symbols by which these ideas are suggested on a printed page. The pupil would pass at once from the sound "cow" to the thing itself—the mental conception of the living and familiar animal; and the printed word would at once become a representative of the animal, and not merely of the sound. But if the sentence were, "I see a zebra," no such process could take place; and the recollection of the sound "zebra," in connexion with the visual symbol, would be all that the child could possibly accomplish. In the latter case, there would be a mere sensational action—a linking together of two sensations in the memory; but, in the former, the sensations would not only be linked together, but would, moreover, be crowned or completed by a distinct conception of the thing signified. It follows that this completion of the act of consciousness will soon become habitual to the learner; and that when, after a time, words representing unknown ideas are introduced into the lesson, these ideas will be sought for by the mind, and an intelligent curiosity will be excited with regard to them. The early word-lessons having always appealed to actual knowledge, the pupil will feel that there is something to be known in connexion with any word that may be strange to him; and hence the sensational impression will always pass on, and excite cerebral activity, either for the contemplation of old ideas, or for the search after new ones. On the contrary, when the first words taught are not understood, it is perfectly natural for the growing brain to remember them without any curiosity, and to fall into the mode of action which it is thus permitted to commence.

As far as very early efforts at learning are concerned, we do not place much confidence in any endeavours, on the part of the teacher, to explain that which the child does not already understand. Such explanations are prone to miss the precise point of difficulty to the pupil, or they are diffuse, or tedious, or in some way wearisome; and, in either case, they fail to arrest the attention, or to rouse the intelligence. For a time varying with differences in respect of natural capacity, we think that occasions for explanation should be avoided, and that the pupil should read only about what he does understand, until the habit of understanding what he reads is established. Then, here and there, but at first sparsely, words and sentences, conveying new ideas, may be introduced. The names of unfamiliar objects will serve this purpose; and the more thoroughly, the more resemblance there is between the objects and others with which the child is acquainted; or, in other words, the smaller the effort of attention and comprehension that is at first required to be made. While the process is still going on, the child's circle of knowledge may constantly be enlarged by means of oral teaching, and by guiding his powers of observation; and that which is gained on one day in this manner, may be made the subject of a reading lesson on the next. The error to be avoided is making the reading lesson itself the vehicle of novelty; for whereas, in oral teaching, only the double association between sound and meaning has to be formed, in reading about new matter the association should be triple in order to be of value. But the exigency of the teacher always demands that two elements out of the three, viz., the appearance, and the sound of the words, should be remembered with more or less of accuracy; and it often happens that this demand monopolizes the available nervous force to provide for sensorial activity, and leaves the most important element of the three, the meaning, wholly unattended to. It is manifest, we think, that the appreciation of a new idea must be less easy than the recollection of an old one; and also that the more difficult the third element in the association is made, the more likely it is to be neglected.

We have already referred, incidentally, to the pleasure which attends the exercise of a new faculty, and to the propriety of making this pleasure a stimulus to exertion. No period of human life displays a more exultant happiness than that in which the infant first discovers his power to walk; and it is impossible to doubt that the early functional activity of the cerebrum is in itself a source of no small gratification. But precisely as the infant becomes wearied, seeks support after a few tottering steps, and would fall if the support were withheld, so the first mental exertion must be adapted to the limits of his strength, and will cease to be pleasurable when those limits are exceeded. On this account we are disposed to consider that Mr. Herbert's rule concerning the duration of each lesson is most valuable and important. In many instances, perhaps, the cerebrum would bear a longer period of application; but in all, whenever its freshness was impaired, sensational learning would take the place of intellectual; and it might often happen that a teacher would fail to recognise the change. In order to avoid all risk, it is certainly the best plan to fix upon a time that shall never be exceeded; and to make this time so short that the learners may practise, in reading, the old-fashioned rule in dietetics, and rise from table with an appetite for more.

Before leaving the subject of the connexion that should exist between instruction and pleasure, we must refer, as briefly as possible, to the practice of those who strive to combine instruction with amusement, in order to point out that, in most cases, the amusement is afforded by a variety and multiplication of sensory impressions, surrounding the idea sought to be conveyed. The picture alphabet may be taken as a type of this class of teaching; and the objections which we have urged against the picture alphabet, apply with increased force to further developments of the system from which it sprung. The object of the teacher should be to produce concentration of mind in the pupil; and amusement, as the etymology of the word implies, tends only to scatter and disturb the thoughts. The two things are, in fact, diametrically opposed to each other; and, as the result of endeavours to combine them, the attention must be given to each by turns. Where, however, the demand made by the lesson upon the faculties of the pupil is neither too great nor too protracted, the need for amusement does not arise; and, under less favourable circumstances, no amount of sensational distraction will restore the tired brain so effectually as a period of complete repose.

We have not left ourselves space to refer to the writing lessons, other than in the most cursory manner. We think they should be governed by the same physiological principles which we have endeavoured to enunciate, and that the pupils should commence by trying to copy words, the meaning of which they understand. We are acquainted with two children whose lessons have yet to begin, but who can write on a slate, in a clear, legible hand, the name and address of every member of the family to which they belong. They have acquired this power simply by having old envelopes given to them as playthings; and either of them, the eldest being under six years old, can tell at a glance to whom any letter left at the house is addressed, and can also recognise the handwriting of any correspondent from whom letters arrive frequently. They have therefore learned to write a limited number of words, but each of those words, instead of being merely a hieroglyphic to be copied, is equivalent to an idea; and the children feel an intelligent pleasure in being able to write down something that represents, to themselves and others, actual and definite knowledge. Moreover, they cannot write these words without cerebration, without recalling their knowledge; and, as long as their copies are enlarged on the same principle, the same result will follow, and purely sensational action will be avoided. A child, however, may fill reams of paper with great A, or with "pot-hooks and hangers," or even with sentences, such as "Governments exercise authority," without his mind being exercised, even in the smallest degree, upon his work.

There can, perhaps, be no greater absurdity than the common practice of causing children to commence their writing lessons in large text, and with capital letters: the cramped position of the tiny hand not allowing sufficient play for the distance which the pen ought to traverse; so that the down-strokes are shaky and feeble, the fingers are tired almost immediately, and the task is rendered unnecessarily irksome. This, however, is a matter of detail that affects only the quality of the performance as a work of art; and that is, therefore, somewhat beyond the limits of our present subject. We desire only to show in what way elementary teaching may be made to develop, rather than to repress, the higher faculties of the intelligence.

It is, perhaps, desirable that we should guard ourselves, in so many words, against the supposition that the foregoing observations are intended to apply to children, however young, who have once mastered the mechanical difficulties of reading, and are able to peruse books with interest and pleasure. We have been speaking solely with reference to the very commencement of the task of instruction; and under an assured belief that the manner in which such commencement is made, will often determine the predominant character of the mental operations through the whole of life. When a child has made sufficient progress to read by himself, the manner in which he has been taught will be conspicuous among the circumstances that determine the degree of pleasure he feels in reading, and the nature of the encephalic action that written symbols excite; and hence, we conceive, the mode of teaching increases in importance, in proportion as other stimulants to mental activity are absent. Among the educated classes, such stimulants are supplied by various circumstances; and teaching, being only one influence among many, modifies the intellectual character in a degree that, while still highly important, may be called comparatively slight. It is among the poor, or rather among the children of the unintelligent and untaught, that the first systematic instruction is the absolute commencement of mental education either for good or evil, and that the choice between sensational and intellectual activity in the pupil rests, almost solely, with the teacher. We need not point out the responsibility which such a power of choice imposes, not so much, perhaps, upon the individual master of any particular school, as upon those in high authority, whose humble instrument he is, and under whose guidance his work in life is carried out. We wish we could hope that our professional brethren generally would regard this work from the point of view that they are able and privileged to reach, and would do justice to the wisdom of the system that combines nine subjects in a scheme of elementary instruction, but makes no provision to secure the intellectual receptivity of those who are fated to be taught. As far as secular knowledge is concerned, such justice would induce us to mourn over wasted energies, and to calculate useless cost; but would permit us the consolation of believing that the losses sustained would be felt only during Time. It is our national duty, however, to teach our children a higher and a better learning; and, in order that we may do so, first to cultivate their minds for its reception. If this cultivation be neglected, while sectarian bigots profanely wrangle; or if it be omitted, in order to facilitate a sensational acquaintance with creeds and formularies, the resulting losses may indeed be felt during Time, but they will only be fully realized in Eternity.

